# Serum NFL discriminates Parkinson disease from essential tremor and reflect motor and cognition severity

**DOI:** 10.1186/s12883-022-02558-9

**Published:** 2022-01-27

**Authors:** Yixian Huang, Caili Huang, Qilin Zhang, Tingting Shen, Jiawei Sun

**Affiliations:** 1 Department of Neurology, The Second Affiliated Hospital of Soochow University, NO. 1055 Sanxiang Road, Suzhou, 215004 China; 2 Department of Psychiatry, Johns Hopkins University School of Medicine, Baltimore, MD 21205 USA; 3 Department of hematology, The Second Affiliated Hospital of Soochow University, NO. 1055 Sanxiang Road, Suzhou, 215004 China

**Keywords:** Parkinson’s disease, Neurofilament light chain, Essential tremor, Biomarker

## Abstract

**Objective:**

To investigate the diagnostic value of serum neurofilament light chain (NFL) for discriminating Parkinson disease (PD) from Essential tremor (ET) and healthy controls, and to evaluate its correlation with some clinical features of PD patients.

**Methods:**

This cross-sectional study measured NFL levels with electrochemiluminescence immunoassay in serum of 146 PD patients, 82 ET patients and 60 age-matched healthy controls. We used multivariate regression analyses to examine whether NFL contributes to PD biomarkers. Disease severity were assessed by Unified Parkinson’s Disease Rating Scale part III (UPDRS III), Hoehn & Yahr (H-Y) stage and Mini-Mental State Examination (MMSE).

**Results:**

Serum NFL levels were significantly higher in PD than in ET and healthy controls (16.6 ± 3.5, 12.2 ± 2.4 and 11.8 ± 2.4 pg/mL, respectively, *p* < 0.001). In patients with PD, serum NFL were markedly increased in patients with advanced H-Y stage and patients with dementia (both *p* < 0.001). The correlation analysis revealed that serum NFL was positively associated with UPDRS III score (*r* = 0.79, *p* < 0.001) and H-Y stage (*r* = 0.86, *p* < 0.001), and negatively correlated with MMSE scores (*r* = − 0.70, *p* < 0.001). Further multivariate regression analyses showed that serum NFL was an independent contributor to motor symptom and cognition severity in PD patients (all *p* < 0.01).

**Conclusions:**

Serum NFL levels were markedly elevated may be a useful clinical biomarker for discriminating PD patients from ET and controls. Serum NFL may serve as a potential blood biomarker for motor and cognition severity of PD.

## Background

Historically, Parkinson’s disease (PD) has been considered primarily as a motor disorder [1]. However, In addition to typical motor symptoms, cognitive impairment is one of the most disabling nonmotor features for PD patients and caregivers, and dementia eventually develops in a significant proportion of PD patients [2]. Essential tremor (ET) is generally regarded as a benign movement disorder with single symptom, and has now been replaced by the view that it may be a heterogeneous neurodegenerative disease [3]. Most PD patients suffer from the tremor dominant type, which has the most symptomatic overlap with ET [4]. Classic ET is characterized by action tremor, which affects the upper limbs in at least 95% of patients. Some PD patients also show postural tremor. Therefore, the differential diagnosis of the two diseases is difficult in some cases. Up to now, no specific blood-biomarker have been identified for differentiating PD patients from ET. A promising candidate of such biomarkers is the neurofilament light chain (NFL).

NFL is a neuronal cytoskeletal protein, which is released into the CSF during neuroaxonal damage and has been shown to be elevated in several neurodegenerative disorders [5–9]. Recently, there are some conflicting reports on the blood level of NFL in PD patients [10]. There is emerging evidence that NFL levels in the blood/CSF of PD patients may be significantly increased in comparison to healthy control subjects and are associated with motor impairment and cognitive decline in patients with PD [11, 12]. We hypothesize that serum NFL may be used to distinguish PD from ET and reflect the disease severity of PD. Therefore, our aim was to study the diagnostic value of serum NFL for differentiating PD from ET, and to evaluate its correlation with motor symptoms and cognitive impairment severity of PD patients.

## Materials and methods

### Participants

All participants were recruited from the Second Affiliated Hospital of Soochow University. This study included 288 participants: 146 patients with idiopatic PD, 82 patients with ET, and 60 healthy controls. All patients with PD met the following inclusion criteria: (1) age 40–80 years old, Han Chinese; (2) All patients were diagnosed independently by two neurologists, according to the United Kingdom PD Society Brain Bank Criteria [13]. patient with atypical parkinsonism such as the multiple system atrophy, progressive supranuclear palsy, and vascular or secondary parkinsonism was excluded. (3) > 2 years diagnosed with PD. (4) receiving stable doses of L-Dopa/carbidopa administered at least 3 times per day for at least 4 weeks before the initial screening visit.

Patients with ET met the diagnostic criteria by the Consensus Statement of the Movement Disorder Society on Tremor [14]. Additionally, 60 age- and sex-matched healthy controls with no history of any neurological or psychiatric diseases were recruited from their spouses and friends of patients with PD at the same period. All subjects were Chinese Han population and at least 8 years of formal education.

We received approval from the ethics committee of the Second Affiliated Hospital of Soochow University. All participants gave written informed consent before inclusion in this study.

### Motor symptom severity assessment

We assessed motor symptom severity of the PD patients using the Hoehn & Yahr (H-Y) stage and the Unified Parkinson’s Disease Rating Scale part III (UPDRS III) score as previously literature described. Which was assessed only during “on” condition [15, 16].

We measured Tremor severity and functional effects of the ET patients using the Fahn-Tolosa-Marin Tremor Rating Scale (TRS) subscales A, B and C score as previously literature described [17]. Each subscale element is rated from 0 to 4 (none to severe tremor) giving a maximum score of 16, 36 and 32 for each subscale.

According to the Jankovic et al.’s criteria (1990) [18], based on the UPDRS, we subdivided PD patients into the tremor-dominant (TD) and postural instability/gait disorder (PIGD) subtypes.

### Diagnostic criteria for PD or ET with mild cognitive impairment and dementia

PD with mild cognitive impairment (PD-MCI) and with dementia (PDD) were diagnosed with the diagnostic criteria of the Movement Disorder Society Task Force [19, 20]. An MMSE score of 26 to 28 was used as a possible PD-MCI diagnostic feature as previously literature described [9, 21]. An MMSE score of ≤25 was used as a possible PDD diagnostic feature as previously literature described [9, 21]. The diagnostic criteria of ET and control with cognitive impairment was consistent with those of PD. Structural MRI and routine laboratory tests were performed for all patients to exclude non-PDD causes of dementia.

### Measurement of NFL

We collected 10 ml peripheral blood from each participant between 8:00 and 9:00 AM prior to clinical assessment and following an overnight fast. Blood samples were centrifuged (2500 g for 15 min) within 1 h of collection and then kept frozen at − 80 °C until assay. Serum NFL concentrations were determined by researchers who were blinded to the clinical diagnosis, and serum samples were transferred onto the single molecule array (Simoa) NF-light Advantage Kit from Quanterix (Lexington, MA), as previously described [10].

### Statistical analyses

Demographic and clinical variables between groups were compared using analysis of variance (ANOVA) for continuous variables, and chi-squared for categorical variables. Since serum NFL levels were normally distributed in in all three groups (Kolmogorove-Smirnov one-sample test, for PD group: *p* = 0.22; for ET group: *p* = 0.14 and for control group: *p* = 0.36), the parametric tests were used. For variables that violated the assumptions of normality, the groups were compared with nonparametric Mann-Whitney U test (for 2 groups) or Kruskal-Wallis test (for > 2 groups). We compared NFL levels among the three groups using ANOVA, and Fisher’s least significant difference (LSD) test was performed for Bonferroni post-hoc pairwise comparisons. Where there was a significance in ANOVA, the effect of sex, age, education, BMI and clinical variables was tested by adding these variables to the analysis model as covariates. To determine the diagnostic accuracy, a receiver operating characteristic (ROC) curve was constructed to determine the area under the curve (AUC) and the values of sensitivity and specificity with 95% confidence interval (CI), and Youden index was determined (sensitivity + specificity - 1.0) to find the optimal cutoff value. Relationships between variables were assessed with Pearson correlation and Spearman Rank correlation, where appropriate. Bonferroni corrections were applied to each test to adjust for multiple testing. A multivariate regression analyses was used to assess association of serum NFL with UPDRS III, H-Y and MMSE while adjusting for potentially confounding demographic and clinical variables, including sex, age, education, BMI, alcohol, age of onset and duration of disease. Effect sizes (0.2 = small effect, 0.5 = medium effect, 0.8 = large difference effect) were calculated using Cohen’s d method for the two-way comparisons and represented the mean difference, in standard deviation units, between the groups of interest. The SPSS Statistics (version 19.0, IBM Corp., Armonk, NY, USA) was utilized for all statistical analyses. All data are expressed as mean and standard deviation (mean ± SD). All *p*-values of < 0.05 was considered significant.

## Results

### Clinical characteristics of participants

The study included 288 participants: 146 patients with PD, 82 patients with ET, and 60 were healthy controls. Demographic and clinical information and serum NFL concentrations of all participants are shown in Table 1. The three groups were matched for age, sex and education. PD patients and ET patients were not significantly different in body mass index (BMI), age of onset and disease duration (all *p* > 0.05). The TRS score of ET patients was 33.2 ± 16.7. The MMSE scores were significantly lower in patients with PD (28.4 ± 2.9) than in patients with ET (29.4 ± 1.4) and in healthy controls (29.8 ± 0.4) (both *p* < 0.01; effect sizes = 0.44 and 0.68, respectively; Kruskal-Wallis test; Table 1). Table 1 shows the mean H-Y stages (2.5 ± 1.0) and UPDRS part III scores (23.2 ± 8.9) during the “on” condition.

**Table 1:**
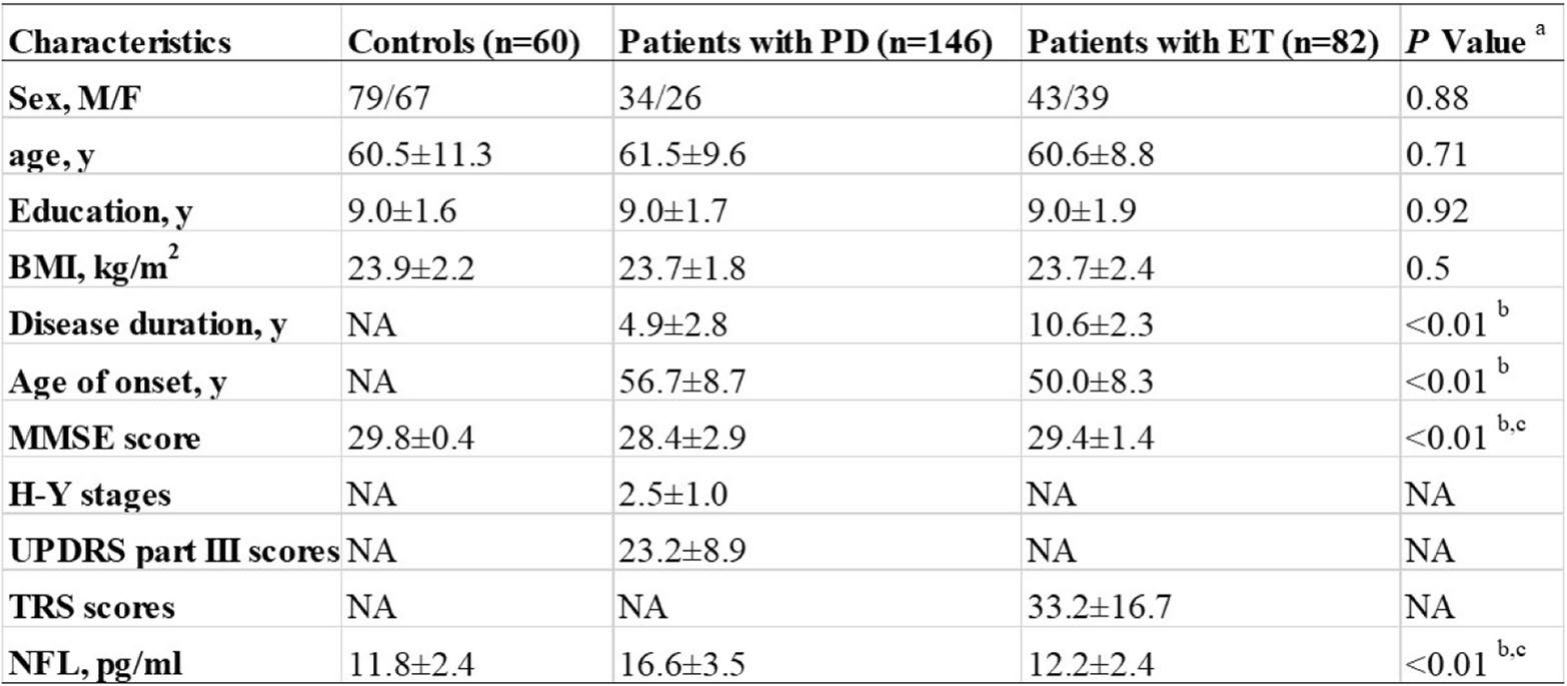
Clinical characteristics and serum NFL levels of participants

Abbreviations: *BMI* body mass index; *ET* essential tremor; *H-Y* Hoehn & Yahr; *MMSE* Mini-mental state examination; *NA* not available; *NFL* neurofilament light chain; *PD* Parkinson disease; *UPDRS* united Parkinson’s disease rating scale

Values are expressed as the mean ± SD

^a^Parameters were analyzed with analysis of variance using Bonferroni as post hoc test in the case of normal distribution of data. For variables that did not display a normal distribution, data were compared with the Kruskal-Wallis test for comparison of multiple groups, or Mann-Whitney U test for comparison of 2 groups. Sex was analyzed using χ2 test

^b^Differences were found between PD vs ET

^c^Differences were found between PD vs control

### Serum NFL concentrations in PD and ET

**Fig. 1:**
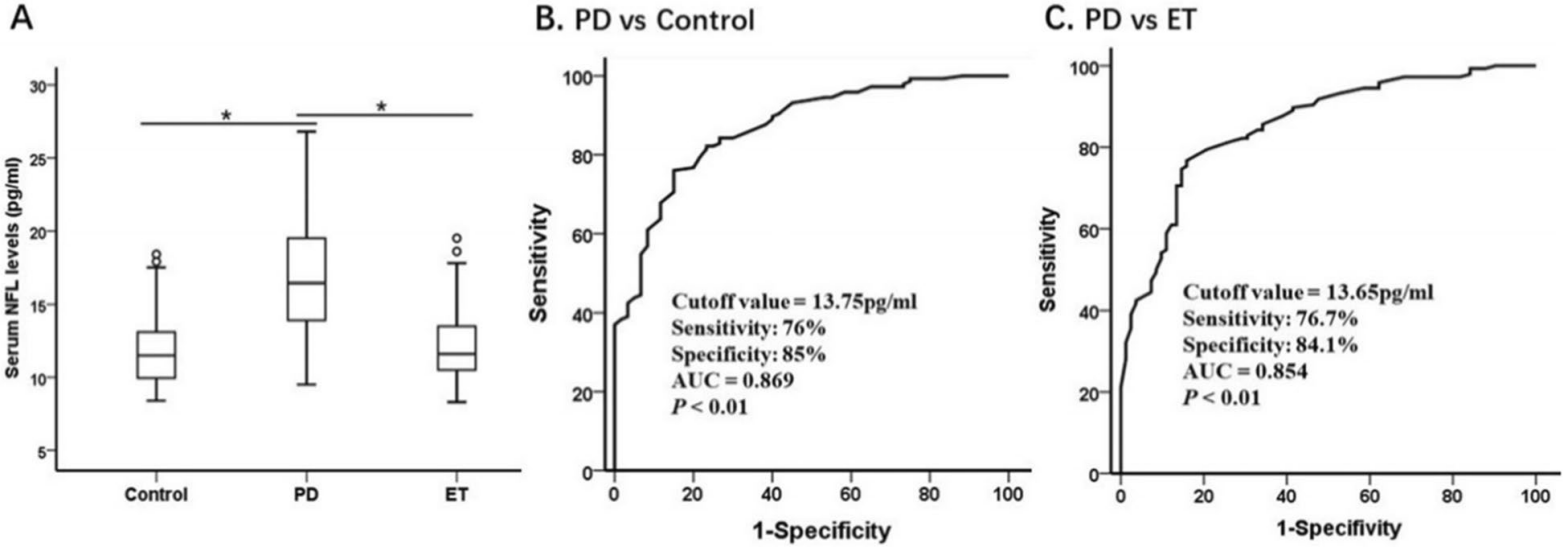
Serum NFL highly discriminates PD from ET and controls. Serum NFL concentration in healthy controls, patients with PD and patients with ET and diagnostic accuracy. (**A**) Mean serum NFL level was significantly elevated in PD compared to ET and nonneurologic healthy controls. Mean levels were shown with SD; *p < 0.01. (B, C), Receiver operating characteristic curve analyses for differentiating between (**B**) patients with PD and age-matched healthy controls and (C) patients with PD and those with ET. AUC = area under the curve; ET = Essential tremor; NFL = neurofilament light chain; PD = Parkinson disease

To determine whether older age might influence serum NFL levels, we examined the correlation between the age of the subjects and NFL levels. Consistent with a previous report, higher serum NFL levels were associated with older age in healthy controls, patients with PD and patients with ET (control: r = 0.30, 95% CI 0.05–0.52, *p* = 0.02; PD: *r* = 0.25, 95% CI 0.07–0.42, *p* = 0.002; ET: r = 0.30, 95% CI 0.13–0.46, *p* = 0.006; Pearson correlation analysis). The serum NFL concentrations were significantly higher in patients with PD (16.6 ± 3.5 pg/ml) than in patients with ET (12.2 ± 2.4 pg/ml) and in healthy controls (11.8 ± 2.4 pg/ml) (both *p* < 0.01; effect sizes = 1.47 and 1.60, respectively; Table 1 and Fig. 1A). As shown in Table 2, PD patients were classified as having the TD and PIGD motor subtypes. There were no significant differences in demographic and NFL level variables in PD patients (all *p* > 0.05). The ROC analysis showed that a serum NFL cutoff value of 13.75 pg/ml had a sensitivity of 76% and a specificity of 85% for distinguishing between PD and healthy controls, with an area under the curve of 0.869 (Fig. 1B). Because serum NFL was higher in patients with PD than in patients with ET (*p* < 0.01, Fig. 1A), the serum NFL cutoff value of 13.65 pg/ml had a 76.7% sensitivity and a 84.1% specificity for distinguishing between PD and ET, with an area under the curve of 0.854 (Fig. 1C).

**Table 2:**
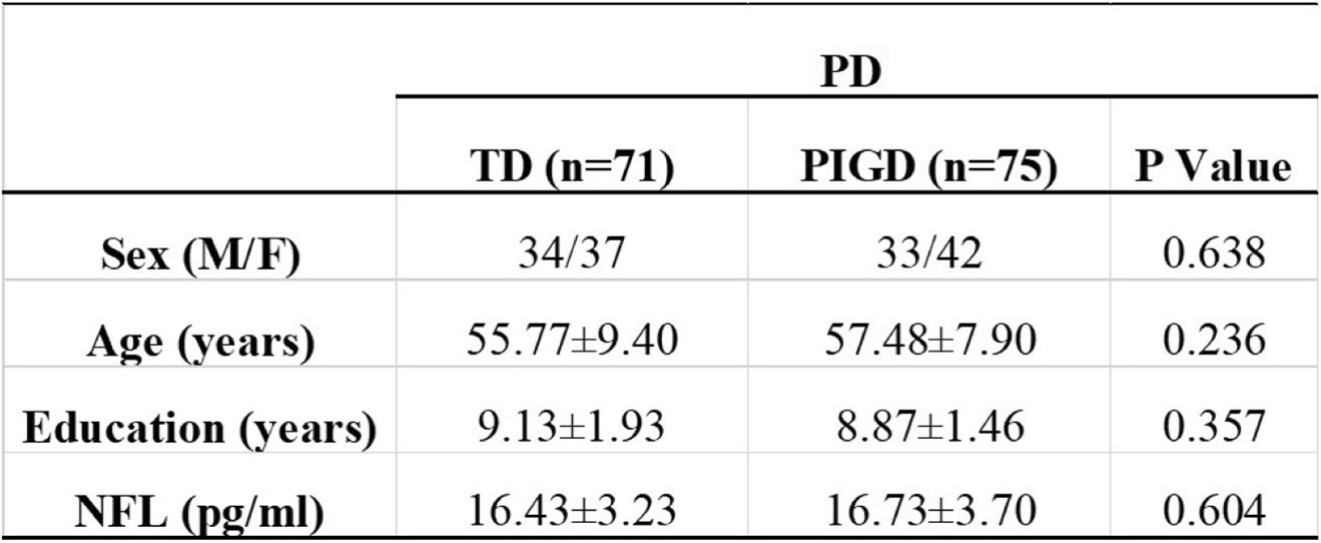
Comparison between TD-PD and PIGD-PD motor subtypes on demographic details and NFL variables

Note: Data are presented as the mean ± standard deviation (Mean ± SD)

Abbreviations: *NFL* neurofilament light chain; *PD* Parkinson disease; *PIGD* postural instability/gait disturbances dominant; *TD* tremor dominant

Parameters were analyzed with analysis of variance using Bonferroni as post hoc test in the case of normal distribution of data. For variables that did not display a normal distribution, data were compared between TD-PD and PIGD-PD groups by Mann-Whitney U test. Sex was analyzed using χ2 test.

### Serum NFL levels were associated with motor severity and cognitive status in PD

**Fig. 2:**
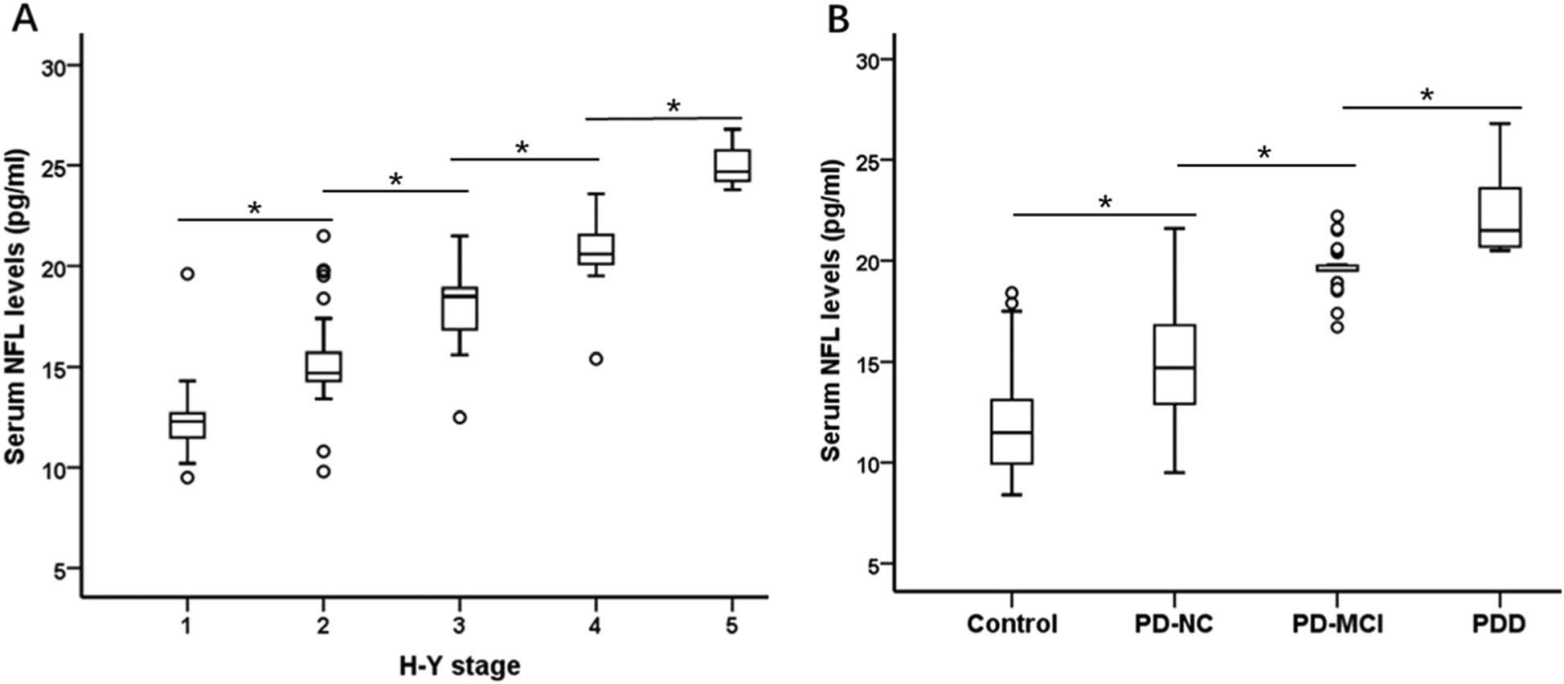
Serum NFL in PD according to motor and cognition severity. (**A**) Mean serum NFL level was significantly elevated in patients with PD who displayed severe motor disorder based on the H-Y stage (*p* < 0.01). (**B**) Mean serum NFL level was markedly higher in patients with PDD compared to PD-MCI, PD-NC and control. Mean levels are shown with SD; *p < 0.01. H-Y = Hoehn & Yahr; MCI = mild cognitive impairment; NFL = neurofilament light chain; PD = Parkinson disease; PDD = PD with dementia; PD-NC = PD with normal cognition

Motor disorder is the most typical dysfunctionis of PD patients. In assessing the motor symptom severity of PD, we observed an rose in serum NFL levels with the increasing motor disorder as reflected by the H-Y stage (Fig. 2A). The Specific serum NFL concentrations were 12.2 ± 1.8, 15.1 ± 2.1, 18.0 ± 1.5, 20.6 ± 1.5, and 25.1 ± 1.5 pg/ml for H-Y stage I through V (*n* = 27, *n* = 46, *n* = 47, *n* = 23, *n* = 3), respectively (F = 104.1, *p* < 0.001). Cognitive impairment is one of the most disabling nonmotor features of PD. We then examined the relationship between serum NFL concentrations and various levels of cognitive function among PD patients and healthy controls. We observed that serum NFL significantly increased as the cognitive impairment severity increased (Fig. 2B). The specific NFL concentrations were 11.8 ± 2.4, 15.0 ± 2.6, 19.6 ± 1.1, and 22.2 ± 1.9 pg/ml for the control, PD with normal cognition, PD-MCI, and PDD groups (*n* = 60, *n* = 102, *n* = 31, *n* = 13), respectively (F = 114.8, *p* < 0.001).

**Fig. 3:**
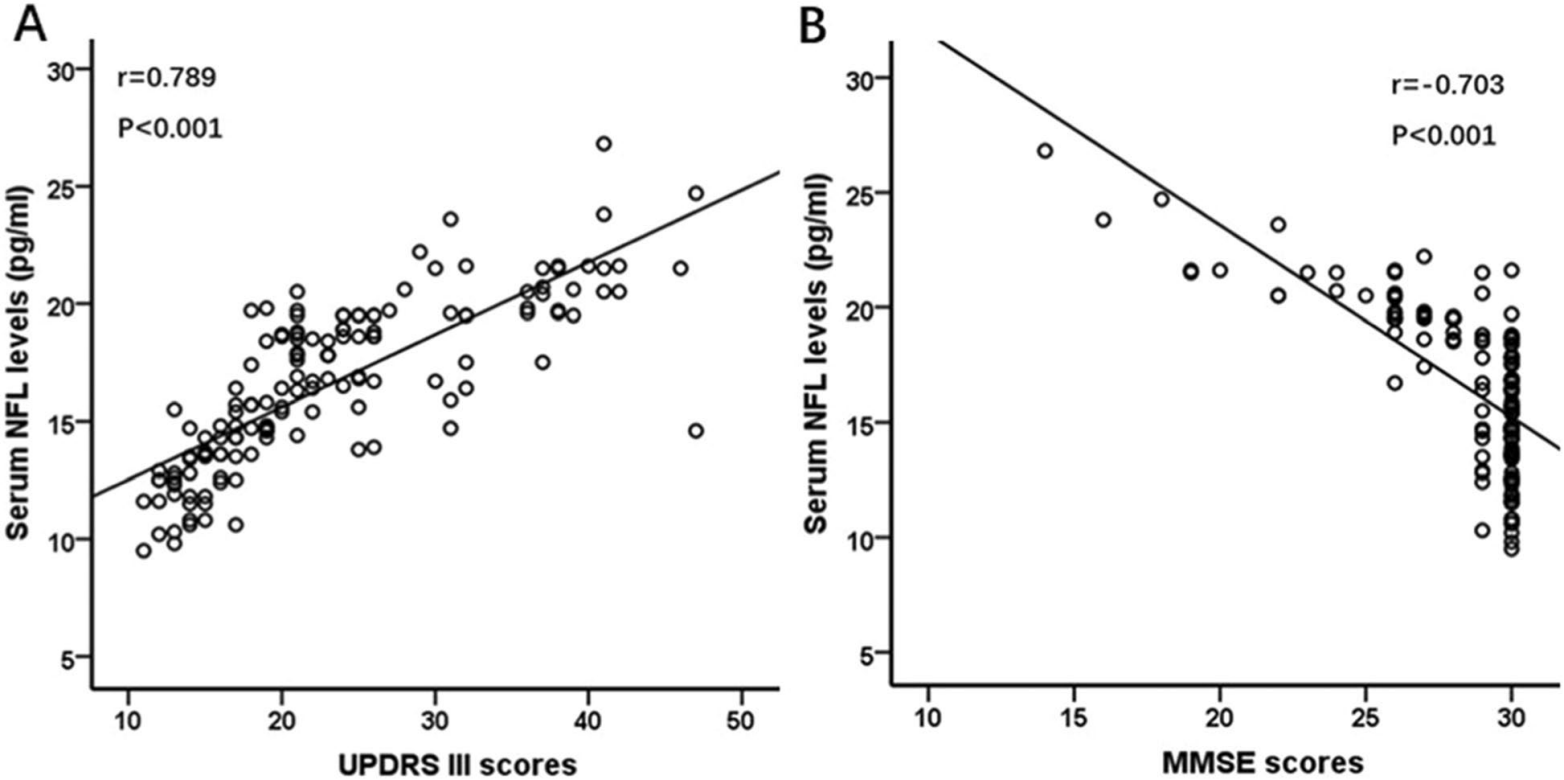
Serum NFL was associated with motor and cognition severity in PD. Correlation analysis showed that (**A**) serum NFL levels were significantly positively correlated with UPDRS III scores (*p* < 0.001), (**B**) serum NFL levels were markedly negatively correlated with MMSE scores (*p* < 0.001). MMSE = Mini-Mental State Examination; NFL = neurofilament light chain; PD = Parkinson disease; UPDRS III = Unified Parkinson’s Disease Rating Scale part III

In patients with PD, correlation analysis showed that serum NFL levels were significantly positively correlated with motor symptom severity, measured with UPDRS III score and H-Y stage (UPDRS III score: r = 0.79, 95% CI 0.70–0.86, *p* < 0.001; Bonferroni corrected *p* < 0.01; Pearson correlation analysis; H-Y stage: r = 0.86, 95% CI 0.78–0.91, *p* < 0.001; Bonferroni corrected p < 0.01; Spearman correlation analysis) (Fig. 3A, Fig. 2A). At the same time, serum NFL levels were significantly negatively correlated with MMSE scores in PD patients (MMSE scores: r = − 0.70, 95% CI − 0.63 to − 0.77, *p* < 0.001; Bonferroni corrected p < 0.01; Pearson correlation analysis) (Fig. 3B).

Further multivariate regression analysis showed that serum NFL was an independent contributor to motor symptom and cognition decline severity in PD patients (H-Y stage: t = 8.75, VIF = 2.93, *p* < 0.001; UPDRS III score: t = 3.17, VIF = 2.92, *P* = 0.002; MMSE score: t = − 4.81, VIF = 1.73, *p* < 0.001), while adjusting for potentially confounding demographic and clinical variables, including age, sex, education, BMI, age of onset, and disease duration. Our data has been normalized and there was no multicollinearity.

## Discussion

In this study, we aimed to analyze whether serum NFL levels could be used as a biomarker for discriminating PD from ET and controls and reflect the motor and cognition severity of PD. For this purpose, we measured NFL concentrations in the serum of patients with PD, ET and age-matched healthy controls. Our results demonstrated that serum NFL levels were significantly increased in PD than ET and controls and discriminated PD from ET and controls with high accuracy levels. Correlation analysis showed that serum NFL was positively associated with UPDRS III score and H-Y stage, and negatively correlated with MMSE scores. Further multivariate regression analysis confirmed that serum NFL was an independent contributor to motor symptom severity and cognition decline in patients with PD, while adjusting for potential confounders, including age, sex, education, BMI, age of onset, and disease duration. Altogether, these findings suggested that increased serum NFL may serve as a surrogate biomarker of motor and cognition severity in PD and discriminated PD from ET and controls.

Abnormal neuronal cytoskeletal proteins is one of the major pathological mechanisms of several neurological disorders [9, 22]. NFL is abundant in axons, thus release of NFL likely occurs after neuroaxonal damage with subsequent release into blood [23, 24]. Recent several studies showed that NFL has become a potential biomarker in PD [9, 25]. Some authors have found NFL levels were elevated in PD patients compared to healthy controls [9, 11], many others have observed no differences between these two groups of subjects [12, 26], but the conclusions were based either on a smaller number of PD patients or involved patients with less advanced PD. So far, the correlation between NFL levels and PD patients remains unclear. Clinically, most PD patients suffer from the tremor dominant type with a most symptomatic overlap with ET [4]. ET is a most prevalent age-related neurological diseases. The precise pathogenesis remains to be elucidated. Recent studies using western blot analysis found no difference in NFL protein expression between the ET patients and controls [27]. Our findings demonstrated that serum NFL levels were markedly elevated in PD compared to ET or controls and discriminated PD from ET and control with high accuracy. The ROC analysis showed that the serum NFL had a sensitivity (76.7%) and a 84.1% specificity (AUC 0.85) for distinguishing between patients with PD and ET. These results suggested that serum NFL may be a useful clinical biomarker for differentiating PD patients from ET and healthy controls.

In the present study, the three groups of subjects were matched for age, sex and education. we found that higher serum NFL levels were associated with older age in three groups, regardless of disease status. This result was consistent with those previously reported [28]. Nonetheless, the reason for this association between age and serum NFL levels remains to be elucidated. Presumably, it could be due to age-related bloodstream clearance decreases with aging or brain hypometabolism, leading to some degree of axonal degeneration and NFL release into bloodstream [23–25].

Motor disorder is the most typical dysfunctions of PD patients. When the various levels of motor ability among patients with PD were considered, serum NFL level showed a positive correlation with motor symptom severity, measured with H-Y stage and UPDRS III score. Interestingly, we observed an rose in serum NFL levels with the increasing motor disorder as reflected by the H-Y stage I through V. Cognitive decline is one of the most disturbing nonmotor features of PD. When the various levels of cognitive ability among patients with PD and controls were considered, serum NFL showed a negative correlation with MMSE score, a clinical measure of global cognitive function in PD. It is interesting that serum NFL levels rose with increasing cognitive decline as reflected by lowest levels MMSE score in participants with PDD through highest MMSE score in healthy controls. These findings were consistent with a recent study published by Lin et al. [9], they found a positive correlation between plasma NFL levels and PD severity assessed in H-Y stage, UPDRS III score and MMSE score. Altogether, our results suggested that, in addition to the diagnostic value of serum NFL levels for differentiating PD from ET and controls, serum NFL might be a biomarker for motor symptom and cognition severity in PD patients.

The current study has some limitations. First, the study design was cross-sectional, which limits causal inference between NFL and clinical features of PD. Second, although we enrolled patients with PD and ET had been diagnosed for more than 2 years, they were diagnosed based on clinical criteria and not a pathological examination, especially for PDD patients. Third, we did not collect some more important clinical data, such as family history, coffee intake, levodopa equivalent dose (LED) calculation, hypertension, diabetes, coronary heart disease and other information, which may also affect the clinical symptoms or serum NFL in PD patients. Fourth, our clinical trial population was entirely han chinese and lacked ethnic diversity. Fifth, the relatively small sample size of each group is a concern. We performed a longitudinal follow-up study in conjunction with neuroimages comparison of PD, ET and controls, which may serve as supplemental evidence for future clinical use of serum NFL as a diagnostic biomarker.

## Conclusion

In conclusion, our findings suggest that markedly elevated serum NFL levels may be a useful clinical biomarker for differentiating PD patients from ET and controls. Furthermore, we found a correlation of serum NFL with disease severity in terms of motor and cognitive performance in PD patients. As such, elevated serum NFL may serve as a potential blood biomarker of motor and cognition severity in PD. A future clinical trial with a larger longitudinal follow-up studies that incorporate other biomarkers such as α-synulcein and neuroimages are needed to validate whether blood NFL may be used to predict PD progression.

## Data Availability

The datasets used and/or analyzed during the current study are available from the corresponding author on reasonable request.

## Abbreviations

AUC: Area under the curve
BMI: Body mass index
CI: Confidence interval
ET: Essential tremor
H-Y: Hoehn & Yahr stage
LED: Levodopa equivalent dose
MCI: Mild cognitive impairment
MMSE: Mini-Mental State Examination
NFL: Neurofilament light chain
PD: Parkinson disease
PDD: PD with dementia
ROC: Receiver operating characteristic curve
Simoa: Single molecule array
UPDRS III: Unified Parkinson’s Disease Rating Scale part III
